# Inflammatory Response and Activation of Coagulation after COVID-19 Infection

**DOI:** 10.3390/v15040938

**Published:** 2023-04-10

**Authors:** Anna Glória Fonseca Teodoro, Wellington Francisco Rodrigues, Thais Soares Farnesi-de-Assunção, Anna V. Bernardes e Borges, Malu Mateus Santos Obata, José Rodrigues do Carmo Neto, Djalma A. Alves da Silva, Leonardo E. Andrade-Silva, Chamberttan S. Desidério, Juliana C. Costa-Madeira, Rafaela M. Barbosa, Andrezza C. C. Hortolani Cunha, Loren Q. Pereira, Fernanda Bernadelli de Vito, Sarah Cristina Sato Vaz Tanaka, Fernanda R. Helmo, Marcela Rezende Lemes, Laís M. Barbosa, Rafael O. Trevisan, Fabiano V. Mundim, Ana Carolina M. Oliveira-Scussel, Paulo Roberto Resende Junior, Ivan B. Monteiro, Yulsef M. Ferreira, Guilherme H. Machado, Kennio Ferreira-Paim, Hélio Moraes-Souza, Carlo José Freire de Oliveira, Virmondes Rodrigues Júnior, Marcos Vinicius da Silva

**Affiliations:** 1 Department of Immunology, Microbiology and Parasitology, Federal University of Triângulo Mineiro, Uberaba 38025-350, Brazil; 2 Postgraduate Program in Physiological Sciences, Federal University of Triângulo Mineiro, Uberaba 38025-200, Brazil; 3 Laboratory of Hematological Research of the Federal University of Triângulo Mineiro and Regional Blood Center of Uberaba—Hemominas Foundation, Uberaba 38025-440, Brazil; 4 UNIMED São Domingos Hospital, Uberaba 38025-110, Brazil; 5 José Alencar Gomes da Silva Regional Hospital, Uberaba 38060-200, Brazil; 6 Mário Palmério University Hospital, Uberaba 38050-501, Brazil

**Keywords:** COVID-19, thrombosis, IL-10, inflammatory response

## Abstract

SARS-CoV-2 (COVID-19) infection is responsible for causing a disease with a wide spectrum of clinical presentations. Predisposition to thromboembolic disease due to excessive inflammation is also attributed to the disease. The objective of this study was to characterize the clinical and laboratory aspects of hospitalized patients, in addition to studying the pattern of serum cytokines, and associate them with the occurrence of thromboembolic events. Methodology: A retrospective cohort study with 97 COVID-19 patients hospitalized from April to August 2020 in the Triângulo Mineiro macro-region was carried out. A review of medical records was conducted to evaluate the clinical and laboratory aspects and the frequency of thrombosis, as well as the measurement of cytokines, in the groups that presented or did not present a thrombotic event. Results: There were seven confirmed cases of thrombotic occurrence in the cohort. A reduction in the time of prothrombin activity was observed in the group with thrombosis. Further, 27.8% of all patients had thrombocytopenia. In the group that had thrombotic events, the levels of IL1b, IL-10, and IL2 were higher (*p* < 0.05). Conclusions: In the studied sample, there was an increase in the inflammatory response in patients with thrombotic events, confirmed by the increase in cytokines. Furthermore, in this cohort, a link was observed between the IL-10 percentage and an increased chance of a thrombotic event.

## 1. Introduction

In late 2019 and early 2020, the world was confronted with the outbreak of a new disease, a viral infection due to the coronavirus that causes Severe Acute Respiratory Syndrome (SARS-CoV-2) [1]. SARS-CoV-2 is responsible for the coronavirus disease 2019 (COVID-19) pandemic. COVID-19 is a complex disease with a wide spectrum of clinical presentations, ranging from asymptomatic infection to severe respiratory failure with multi-organ failure and death [2].

Studies have pointed out that SARS-CoV-2 is cytopathic and can cause significant damage to the lungs, as evidenced in pathological examinations [3]. Another common finding in patients with COVID-19 is the presence of severe endothelial injury with rupture of the endothelial cell membrane and the presence of microthrombi in the alveolar capillaries [4]. These observations clearly point to the identification of a coagulopathy associated with COVID-19, which may contribute to thrombosis, multi-organ damage, severity, and mortality. In COVID-19, the amplitude of the immune system activated varies between individuals. The exacerbated activation of cytokines, called “cytokine storm”, is believed to play a crucial role in the pathogenesis of severe cases of COVID-19 [3]. Most severe cases of COVID-19 with respiratory distress syndrome were associated with high systemic levels of IL-1β, TNF-α, and IL-6 [1].

Severe SARS-CoV-2 infections are characterized by an acute inflammatory response and are almost invariably accompanied by changes in the coagulation system. In general, following injury by a microorganism, immune cells are recruited, and pro-inflammatory cytokines are generated; these cytokines are mediators of coagulation activation [5].

The pathological profile of thrombosis due to COVID-19 is yet to be fully clarified; however, a significant finding is that coagulation alterations are primarily mediated by platelet activation and intrinsically related to virus-mediated endothelial inflammation [6]. Thrombocytopenia is a common laboratory abnormality in critically ill patients and is associated with poor clinical outcomes, including death [2]. In a meta-analysis of 31 observational studies involving 7613 participants, the authors demonstrated an association between severe COVID-19 and thrombocytopenia greater than 3 times, a statistically significant relationship (OR = 3.46; CI = 1.72 to 6.94) [7].

It is known that IL-1β, IL-6, and IL-8 are involved in most acute inflammatory diseases. Acute infections can alter hemodynamics, as well as the coagulation and fibrinolytic systems, in such a way as to precipitate ischemic events, with thromboembolism related to inflammatory processes [5]. IL-6 is responsible for one of the main mechanisms by which inflammation activates coagulation by inducing tissue factor expression [5]. Fibrinogen, a biomarker and clotting factor, indicates the presence of inflammation. Fibrinogen and fibrin degradation products stimulate the expression of IL-6 mRNA and proteins, TNF-α, demonstrating a close correlation between the immune system and the coagulation system in the pathogenesis of several diseases [8].

In COVID-19, when the virus causes tissue damage, immune cells are recruited, and pro-inflammatory cytokines, which are mediators of coagulation activation, are generated [5]. High expression levels of IL-1β and IFN-γ have been detected in patients with COVID-19 [9]. Notably, other studies have also indicated that patients with COVID-19 have elevated levels of cytokines secreted by Th2 cells (such as IL-4 and IL-10), which normally inhibit the inflammatory response [9]. Increased levels of plasma concentration of IL-1B, IL1RA, IL7, IL8, IL9, IL10, IFNγ, and TNFα are noted in both intensive care unit (ICU) and non-ICU patients compared to healthy adults [10]. The rate of increase in cytokine levels varies depending on the period of COVID-19 infection, characteristics of the viral strain, and the individual characteristics of patients.

A study showed a frequency of acute thrombotic events in 5.2% of patients hospitalized with COVID-19; thrombotic events occurred in 8.8% of ICU patients [11]. A meta-analysis, comprising 28,173 patients with COVID-19 (of which 94% were hospitalized), estimated an in-hospital prevalence of venous thromboembolism (VTE) of 14.1%. Given the high mortality, especially in ICU patients with COVID-19, the competing risk of death might lead to an underdiagnosis of VTE [12]. For the quantitative assessments of the aforementioned meta-analysis, the authors disregarded all studies with a high risk of bias (*n* = 20), attributing a low risk of bias to the studies eligible for the meta-analysis, using the Joanna Briggs Institute Critical Appraisal Checklist and the evaluation of publication bias by funnel plot. Data for GRADE were not presented.

In this context, this study evaluated serum samples obtained from patients with different clinical forms, seeking to determine factors that contribute to an exacerbated and unregulated production of cytokines and markers of alteration in the coagulation system in patients with COVID-19. A clinical laboratory evaluation of patients hospitalized in the Triângulo Mineiro/MG macro-region was carried out, enabling description of the characteristics of the immune system and coagulation in COVID-19.

## 2. Materials and Methods

### 2.1. Patients and Samples

Initially, a sample of 100 patients with clinical symptoms suggestive of COVID-19 was selected, considering the clinical and epidemiological diagnostic criteria of the disease. As an epidemiological criterion, we considered cases of individuals who had contact with a patient with a positive laboratory test for COVID-19 in the last 7 days. The study included 97 patients with clinical and epidemiological characteristics of COVID-19, as well as some laboratory or radiological examination results suggestive of the disease.

A serological test for COVID-19 or RT-PCR was the laboratory test considered for diagnosis, with 79 patients testing positive. In addition, a chest tomography with a ground-glass appearance was the radiological examination used as a criterion. Of the selected patients, the characteristic tomographic aspect was identified in 88 cases. Three patients with negative laboratory tests for SARS-CoV-2 and CT scans that were not characteristic of the disease were excluded from the study. In addition, all patients under 18 years of age and those who did not sign the informed consent form to participate in the study were excluded. Patients with previously known coagulation disorders were also excluded.

Collections were carried out from April to August 2020. Considering that the vaccination process against COVID-19 began in January 2021, the samples are predominantly from the period of the first wave. The first epidemic wave was characterized by the circulation of multiple strains of SARS-CoV-2. The recruitment of participants for this study was carried out in Uberaba-MG, Brazil, specifically in the places established for outpatient and hospital care of cases of COVID-19 in the Triângulo Mineiro macro-region (Emergency Care Units, Regional Hospital and Clinical Hospital/Federal University of Triângulo Mineiro).

Considering the collection period and that the first wave of COVID-19 occurred between February and July 2020, the samples are predominantly from the period of the first wave. In the initial period of 2020, relatively few tests were carried out to identify the various strains [13]. The first epidemic wave was characterized by the circulation of multiple strains of SARS-CoV-2. At the end of October 2020, variants such as Zeta, Alpha, and Gamma were identified [14]. This research was carried out with data prior to the identification of these variants.

### 2.2. Analysis of Records and Clinical History of Patients

A systematic review of the medical records of the 97 selected patients was carried out, analyzing the clinical repercussions of the disease and the frequency of thrombotic events. Deep venous thrombosis, pulmonary thromboembolism, acute myocardial infarction, ischemic stroke, and arterial embolism were the thrombotic events considered. Only confirmed events that had well-founded clinical suspicion associated with a confirmatory diagnostic imaging method or macroscopic confirmation of thrombosis during a surgical procedure were considered.

The clinical condition of the patients was classified as mild, moderate, or severe. Those who required mechanical ventilation and intensive care in the ICU were considered serious or critical. All those who needed some type of oxygen support for more than 24 h were classified as moderate.

### 2.3. Percentage of Cytokines and Chemiokines

Peripheral blood samples were collected in a tube without anticoagulant and containing separated gel from where the sera were stored. Cytokines, IL-2, IL-4, IL-1β, TNF-α, IL-6, IL-8, IL-10, IL-12, IL-17, and IFNy were measured using the Cytometric Bead Array (CBA) technique (BD Biosciences, NJ, USA), according to the manufacturer’s instructions. The samples were analyzed in a FACSCalibur flow cytometer (BD Biosciences) using the CellQuest Pro program (BD Biosciences). The concentration of cytokines was calculated based on a standard curve composed of samples with known concentrations. After the acquisition of data from samples and recombinant cytokines, they were analyzed using FCAP Array 2.0 software (Soft Flow, PÉCS, HUNGARY).

### 2.4. Analysis of the Coagulation System

Data contained in routinely requested laboratory tests, such as blood count, platelet count, prothrombin activity time (PAT), and activated partial thromboplastin time (APTT), were analyzed. A limitation of the study was the lack of laboratory tests for all patients in the sample, since the research was carried out by reviewing the medical records. Analyses were performed according to available data on 52 patients with PAT and 44 with APTT. Platelet counts were also stratified, and values between 150,000 and 450,000/mL were considered normal. Thrombocytopenia was considered when the values were less than 150,000/mL, and thrombocytosis was considered when the values were greater than 450,000/mL. Thrombocytopenia was graded mild when the values were between 100,000 to 150,000/mL, moderate when the values were between 50,000 and 100,000/mL, and severe when the values were less than 50,000/mL [15].

### 2.5. Statistical Analysis

Data were tabulated in Microsoft^®^ Excel and analyzed using IBM SPSS Statistics 23 (IBM Corp., Armonk, NY, USA). After extracting the data to the spreadsheet, the steps for verification and “cleaning” of the database were executed. Continuous variables were checked for distribution using the Shapiro–Wilk test, and homoscedasticity was assessed using the Levene test. Welch correction was used for cases of unequal variances. The unpaired *t*-test was used for evaluations with Gaussian distributions and without differences in variances, and the Mann–Whitney test was used for non-parametric comparisons. The effect size was determined by Cohen’s d for the application of the parametric test and the biserial point correlation coefficient for the non-parametric test. The chi-square test was used to assess possible associations between categorical variables. A binomial logistic regression model was used to assess the relationship between cytokines and thrombotic events, and the effect size was estimated by the odds ratio with the respective confidence interval (95%). The G*Power version 3.1.9.7 program was used to determine the power for the inferences of the logistic regression model using post hoc analysis. A significance level of 5% was considered in all analyses (R Core Team, 2020; Arango, 2001).

### 2.6. Ethical Aspects

This project is part of a set of projects developed at the Federal University of Triângulo Mineiro (UFTM) to counter the onslaught of the COVID-19 pandemic. In particular, this proposal is part of a large project titled “Assessment of the immune response to COVID-19 infection in patients treated in Uberaba-MG”, approved by the National Research Ethics Committee. Patients were included in the study after they agreed to participate and signed an informed consent form. Research manuscripts reporting large datasets that are deposited in a publicly available database should specify where the data have been deposited and provide the relevant accession numbers. If the accession numbers have not yet been obtained at the time of submission, the researchers undertake that they will be provided during review. They must be provided at least prior to publication. Interventional studies involving animals or humans, and other studies that require ethical approval, must list the authority that provided approval and the corresponding ethical approval code.

## 3. Results

### 3.1. Population Analysis

According to the eligibility criteria, a total of 97 medical records were selected and evaluated. All cases were related to the diagnostic hypothesis for COVID-19 through the initial clinical parameters. The frequencies for females and males, as well as possible discrepancies between genders regarding age rates and length of stay, were evaluated. A difference of 15.46% was observed between the frequency of males (57.73%) and females (42.27%), but these differences were not significant (*χ*^2^ = 2.32; *p* = 0.13). The mean age in years (female: 60.70 ± 18.10 and male: 60.5 ± 15.30) and length of stay in days (female: 13.60 ± 16.80 and male: 10.60 ± 9.14) did not show significant differences between genders (Table 1).

The rate of positive diagnostic tests for COVID-19 was 81.44%. In 88 of the 97 patients selected, the tomographic aspect characteristic of the studied disease was identified, and radiological examination was used as a confirmatory test for COVID-19 (Table 2).

Regarding the clinical presentation of the 97 patients evaluated, it was observed that 53.6% had severe condition, 23.7% had moderate condition, and 22.7% had mild condition. In addition, it was found that a large proportion of hospitalized patients needed some form of oxygen support, with 77.3% of patients using supplemental oxygen through a nasal catheter, oxygen mask, or tracheal intubation. Of the patients who required tracheal intubation, 9% improved with clinical treatment and were extubated (Table 3).

### 3.2. Analysis of Thrombotic Events and Related Laboratory Tests

Confirmed thrombotic events included deep vein thrombosis, pulmonary thromboembolism, acute myocardial infarction, ischemic stroke, and arterial embolism. In the studied sample, seven patients had some type of thrombotic event (Table 4). Of the thrombotic events, there were four cases of pulmonary thromboembolism or deep vein thrombosis, two cases of ischemic stroke, and one case of arterial embolism. The rate of use of anticoagulants was studied, and almost 93% of the analyzed patients were medicated with enoxaparin or heparin during the hospitalization period. Thus, there was a relevant percentage of thrombosis, even when measures were used to prevent these events. In this study, 27 (27.8%) cases of thrombocytopenia (<150,000/mL) were recorded, of which 19 were considered mild, 6 moderate, and 2 severe. The thrombocytosis rate (>450,000/mL) was also evaluated at the rate of 5.1% (Table 4).

Regarding laboratory tests related to coagulation, the prothrombin activity time (PAT) showed less activity in the group with thrombotic events. Despite this difference between the groups not being significant, the biserial point correlation shows that there is a significant difference between the two analyzed groups and that if the sample were larger, it would probably lead to a significant result. The Activated Partial Thromboplastin Time (APTT) did not present significant differences between the groups. In both groups, there are laboratory tests of PAT and APTT with alterations in relation to normality. This was probably due to the complexity of the disease caused by SARS-CoV-2, which can lead to an inflammatory process and changes in clotting factors, regardless of the presence of a thrombotic event (Table 5).

### 3.3. Hematopoietic Line Analysis and Other Laboratory Exams

In the analysis of the blood count of hospitalized patients with COVID-19, it was observed that in cases where there was a thrombotic event, the red blood cell count was significantly lower compared to the group that did not have an event (*p* < 0.050). Patients who had a thrombotic event also showed a reduction in the average values of hemoglobin and hematocrit, but without a significant effect. It is also noteworthy that there were significant differences in the leukocyte, neutrophil, and monocyte counts, which were higher in the group that had a thrombotic event (*p* < 0.05). Regarding other laboratory tests, such as protein C-reactive (PCR), total proteins, and albumin, there was no significant difference between patients with thrombosis and those without thrombotic events (Table 6).

### 3.4. Analysis of Interleukins in the Evidence of Thrombotic Events

Several cytokines have been evaluated in patients with COVID-19 who did or did not have a confirmed thrombotic event. In the group that had thrombotic events, the levels of IL1b, IL-10, and IL2 were significantly higher than in the absence of thrombotic events (*p* < 0.05). Regarding IL-6 and TNF-a, the data showed higher levels of both cytokines in patients with a thrombotic event, but without statistical significance (Table 7).

Finally, after reporting that higher concentrations for interleukins 1β, 2, and 10 were found in patients associated with thrombotic events and with clinical characteristics for COVID-19, the effect size of the association of these cytokines in these patients was verified (Table 7). The elevation of all three interleukins was related to thrombotic events. For each elevation of one IL-1β unit, that is, for each picogram per milliliter increase in IL-1β, the odds ratio for a thrombotic event increased by 0.6% for a patient with clinical characteristics of COVID-19 (CI = 0.999 to 1.012); this percentage increased to 1% when evaluating IL-2 (CI = 0.998 to 1023.00) and IL-10 (CI = 1.001 to 1.012). A significant effect for the evaluated sample was found only for IL-10 (*p* = 0.02) (Table 8). The statistical power of inferences for the presented logistic regression was 5.1% for IL-1β and 5.2% for IL-2 and IL-10 (alpha 5%).

## 4. Discussion

COVID-19 infection can not only cause an exacerbated activation of cytokines, but also increase the frequency of thrombotic events in patients. It is known that inflammatory response and coagulation activation are two important responses in the defense against infection that do not work independently, but cooperate in a complex and synchronous way [16]. It is extremely important for us to better understand how this relationship occurs in SARS-CoV-2 infection in order to establish treatment and diagnostic protocols for the disease.

The population of hospitalized patients with COVID-19 in the Triângulo Mineiro macro-region was analyzed from this perspective. In this study, there were seven confirmed cases of thrombotic events, even in the presence of measures for thromboprophylaxis. The frequency of thrombotic events varies in the literature, as they depend on the diagnostic methods used and the population assessed. Coagulopathy is a common abnormality in patients with COVID-19. One study reported a frequency of up to 27% of thromboembolism in patients with COVID-19 [17]. Another study reported a frequency of 5.2% acute thrombotic events in patients hospitalized with COVID-19, comprising venous thromboembolism (2.7%), acute ischemic stroke (1.2%), and acute myocardial infarction (1.2%). Thrombotic events occurred in 8.8% of patients in the ICU [11].

Platelets are increasingly recognized as mediators of inflammation and immune dysfunction. Patients with COVID-19 are at increased risk of critical illnesses that cause secondary mortality due to an increased inflammatory state. Thus, it is not surprising that platelets contribute to its pathophysiology and play a significant role in the development of complications associated with COVID-19 [2]. It was determined that among 3915 patients with SARS-CoV-2 infection, there was a rate of 20% thrombocytopenia [2]. This information corroborates data from this study, which found a thrombocytopenia rate of 27.8%.

It is known that severe COVID-19 illness is accompanied by reduced erythrocyte turnover, low hemoglobin levels, and increased serum concentrations of total bilirubin and ferritin. Furthermore, the expansion of erythroid progenitors in peripheral blood, along with hypoxia, anemia, and coagulopathies, is highly associated with severity and mortality [18]. In our study, it was observed that in cases where a thrombotic event occurred, the red blood cell count was significantly lower compared to the group that did not present an event (*p* < 0.050), which may suggest an association between the presence of anemia and coagulopathy.

In addition, this study established a relationship between the inflammatory response and the activation of coagulation, showing an increase in the percentages of IL1 β and IL2 in patients with COVID-19 who presented thrombotic events. When the virus causes tissue damage, immune cells are recruited and pro-inflammatory cytokines are generated. These act as mediators of coagulation activation [5]. High expression levels of IL-1 β and IFN-γ have been detected in patients with COVID-19 [9]. The data from this study show higher levels of IL-6 and TNF-a in patients with a thrombotic event, but without statistical significance. It is noteworthy that the serum levels of IL-6 in patients with COVID-19 are positively correlated with the severity of the disease [9].

Furthermore, in patients with thrombotic events, the levels of IL-10, an interleukin usually associated with anti-inflammatory mechanisms, were also higher. Notably, other studies have also indicated that patients with COVID-19 have elevated levels of cytokines secreted by Th2 cells (such as IL-4 and IL-10), which inhibit the inflammatory response [9].

Initially, IL-10 was described as Cytokine Synthesis Inhibitory Factor (CSIF) and considered a part of the Th2 cytokine pattern; however, later it was realized that IL-10 is also produced by late Th1 cells. IL-10 is a potent suppressor of inflammatory effector functions. However, like many other cytokines, it can also promote B cell proliferation, differentiation, and class switching [19]. Some studies have already found that IL-10 is produced by several types of cells, has multiple functions, and can negatively regulate inflammatory effector cells; however, it can also lead to inflammation. This would justify the result of our study, which identified a relevant increase in IL-10.

Hyperinflammation is a potentially fatal condition associated with several clinical disorders characterized by excessive immune activation and tissue damage. Multiple cytokines promote the development of hyperinflammation; however, the contribution of IL-10 remains unclear. A study on hemophagocytic lymphohistiocytosis (HL), a prototypical hyperinflammatory disease, suggests that IL-18 and IL-10 may collectively promote the onset of a hyperinflammatory state [20]. In cases of severe COVID-19, there is an exacerbated acute inflammatory response that is almost invariably accompanied by changes in the coagulation system. Thus, the presence of IL-10 in the hyperinflammation process may contribute to the development of coagulation disorders and thrombotic events.

Nowadays, the participation of IL-10 in pro-inflammatory phenomena is widely accepted. Experiments have already suggested that this cytokine can activate the vascular endothelium, causing the adhesion of leukocytes. Under basal conditions and in the absence of IL-10, endothelial cells do not allow platelet adhesion, but with increasing cytokine concentration, there seems to be a selective and orderly stimulus for platelet adhesion receptors. Studies have indicated that at high percentages, IL-10 may be a key factor in controlling endothelial cell proliferation in a variety of disease processes [21]. This may justify the findings of this study, which indicated a connection between increased IL-10 and thrombosis.

Therefore, although the association found between the increase in IL-10 and the increased chance of a thrombotic event seems somewhat contradictory, this finding can be justified by the redundant action of this interleukin, which can also act as an inflammatory agent in some situations.

## 5. Conclusions

High levels of cytokines, such as IL-2, IL-1b, and IL-10, may be associated with thrombotic complications in patients with COVID-19. In this study, a relationship between increased IL-10 percentage and an increased chance of a thrombotic event was observed. However, as the sample had only seven cases of thrombotic events, other studies must be carried out to clarify this association.

## Figures and Tables

**Table 1:** Data related to sex, age, and length of stay for hospitalized patients with clinical suspicion of COVID-19.

General Characteristics					
**Characteristic**	**Sex**				
	**Female**	**Male**	**Total**	***χ*** **^2^**	***p*-Value**
N	41	56	97	2.32	0.13
%	42.27	57.73	100.00		
	**Age**				
	**Female**	**Male**	**T-student**	**Cohen-d**	***p*-Value**
Md	62.00	60.50	0.12	0.02	0.91
Ẋ	60.70	60.30			
SD	18.10	15.30			
	**Length of stay**				
	**Female**	**Male**	**T-student**	**Cohen-d**	***p*-Value**
Md	7.00	7.00	1.14	0.23	0.26
Ẋ	13.60	10.60			
SD	16.80	9.14			

N = number. % = percentage. Md = medium. Ẋ = average. SD = Standard Deviation.

**Table 2:** Rate of positive laboratory tests and rate of CT scans with characteristic appearance for COVID-19.

**Characteristic**	**Confirmation for COVID-19**			
	**N**	**%**	***χ* ^2^**	***p*-Value**
Negative	18	18.56	38.40	<0.001
Positive	79	81.44		
Total	97	100.00		
**Characteristic**	**Lung ground-glass appearance**			
	**N**	**%**	***χ* ^2^**	***p*-Value**
Negative	9	9.28	64.30	<0.001
Positive	88	90.72		
Total	97	100.00		

N = number. % = percentage. χ^2^ = Chi-square.

**Table 3:** Evaluation of frequencies for classification of severity, oxygen support, and method for oxygen support in hospitalized patients in a hospital in the Triângulo Mineiro region, Brazil.

**Characteristic**	**Severity Rating**			
	**N**	**%**	***χ* ^2^**	***p*-Value**
Mild	22	22.68	18.00	<0.001
Moderate	23	23.71		
Severe	52	53.61		
Total	97	100.00		
**Characteristic**	**Oxygen Support**			
	**N**	**%**	***χ* ^2^**	***p*-Value**
No	22	22.68	29.00	<0.001
Yes	75	77.32		
Total	97	100.00		
**Characteristic**	**Method for oxygen support**			
	**N**	**%**	***χ* ^2^**	***p*-Value**
IOT	42	43.30	69.90	<0.001
Nasal catheter	23	23.71		
Ambient air	22	22.68		
Extubation	9	9.28		
Tracheostomy	1	1.03		
Total	97	100.00		

N = number. % = percentage. *χ*^2^ = Chi-square.

**Table 4:** Description of frequencies for thrombotic events, use of anticoagulants, thrombocytosis, thrombocytopenia, and classification of thrombocytopenia in hospitalized patients with clinical suspicion of COVID-19 in a hospital in the Triângulo Mineiro region, Brazil.

**Characteristics**	**Thrombotic Event**			
	**N**	**%**	***χ* ^2^**	***p*-Value**
No	90	92.78	71.00	<0.001
Yes	7	7.22		
Total	97	100.00		
**Characteristics**	**In use of anticoagulant (heparin)**			
	**N**	**%**	***χ* ^2^**	***p*-Value**
No	7	7.22	71.00	<0.001
Yes	90	92.78		
Total	97	100.00		
**Characteristics**	**Thrombocytosis**			
	**N**	**%**	***χ* ^2^**	***p*-Value**
No	92	94.85	78.00	<0.001
Yes	5	5.15		
Total	97	100.00		
**Characteristics**	**Thrombocytopenia**			
	**N**	**%**	***χ* ^2^**	***p*-Value**
No	70	72.16	19.10	<0.001
Yes	27	27.84		
Total				
**Characteristics**	**Classification of thrombocytopenia**			
	**N**	**%**	***χ* ^2^**	***p*-Value**
Mild	19	70.37	17.60	<0.001
Moderate	6	22.22		
Severe	2	7.41		
Total	27			

N = number. % = percentage. *χ*^2^ = Chi-square.

**Table 5:** Analysis of PAT and APTT laboratory tests in patients hospitalized with COVID-19.

Parameter	Thrombosis	N	Ẋ	DP	U	*p*-Value	BPC
PAT—Activity (%)	Negative	47	86.39	17.90	57.00	0.06	0.51
	Positive	5	76.08	11.55			
PAT—I.N.R	Negative	47	1.14	0.22	56.50	0.06	0.52
	Positive	5	1.29	0.14			
APTT—Relation	Negative	39	1.24	0.31	80.00	0.53	0.18
	Positive	5	1.30	0.23			

**Table 6:** Description and comparison of clinical laboratory parameters among patients with clinical suspicion of COVID-19, confirmed or not for a thrombotic event, assessed in a hospital in the Triângulo Mineiro region, Brazil.

Parameter	Thrombosis	N	Ẋ	SD	U	*p*-Value	BPC
Red Cells (10^6^/mm^3^)	Negative	90	3.88	0.94	173.00	0.04 *	0.45
	Positive	7	3.17	3.09			
Hemoglobin (g%)	Negative	90	11.46	2.91	193.00	0.09	0.39
	Positive	7	9.66	2.09			
Hematocrit (%)	Negative	90	34.73	8.40	224.00	0.21	0.29
	Positive	7	31.11	6.12			
Platelets (10^3^/mm^3^)	Negative	90	265,055.55	104,051.32	292.00	0.75	0.07
	Positive	7	296,142.86	166,208.13			
Leukocytes (/mm^3^)	Negative	90	11,511.74	6567.84	162.00	0.03 *	0.49
	Positive	7	18,562.86	9289.58			
Neutrophils (/mm^3^)	Negative	89	8880.53	5586.20	167.00	0.04 *	0.46
	Positive	7	14,704.96	8352.91			
Rods (/mm^3^)	Negative	89	627.02	1051.94	243.00	0.31	0.22
	Positive	7	665.84	705.51			
Lymphocytes (/mm^3^)	Negative	89	1360.80	873.83	231.00	0.26	0.26
	Positive	7	1597.59	699.12			
Monocytes (/mm^3^)	Negative	89	613.72	389.49	141.00	0.02 *	0.55
	Positive	7	1190.01	699.97			
Basophils (/mm^3^)	Negative	89	1.69	9.15	301.00	0.64	0.03
	Positive	7	0.00	0.00			
PCR (mg/L)	Negative	75	114.33	115.42	157.00	0.55	0.16
	Positive	5	77.62	80.96			
Total Proteins (g/dL)	Negative	51	5.74	0.87	110.00	0.63	0.14
	Positive	5	5.60	0.31			
Albumin (g/dL)	Negative	53	2.97	0.56	114.00	0.26	0.28
	Positive	6	2.73	0.41			

N = number. % = percentage. Ẋ = average. SD = Standard Deviation. U = Mann–Whitney test. BPC = Biserial point correlation. * = statistically significant differences (*p* < 0.05).

**Table 7:** Evaluation of cytokines in relation to thrombotic events for patients with suspected COVID-19 in a hospital in the Triângulo Mineiro region, Brazil.

Parameter (pg/mL)	Thrombosis	N	Ẋ	SD	Test-*t*	*p*-Value	Cohen’s d
IL-12p70	Negative	51	4.98	1.98	−1.62	0.11	−0.84
	Positive	4	6.75	3.61			
IL1-β	Negative	51	22.91	66.37	−2.5	0.02 *	−1.30
	Positive	4	137.52	254.15			
IL-8	Negative	51	1628.58	3548.04	−0.86	0.39	−0.45
	Positive	4	3240.43	4426.78			
IL-17	Negative	51	36.9	68.78	0.07	0.94	0.03
	Positive	4	34.48	35.09			
IFN-γ	Negative	51	83.55	428.78	−0.09	0.93	−0.05
	Positive	4	104.16	185.65			
TNF-α	Negative	51	43.37	202.08	−0.78	0.44	−0.41
	Positive	4	129.09	226.96			
IL-10	Negative	51	39.84	91.59	−3.05	0.004 *	−1.58
	Positive	4	211.84	261.35			
IL-6	Negative	51	601.19	2078.78	−1.06	0.29	−0.55
	Positive	4	1737.08	1763.29			
IL-4	Negative	51	16.75	17.46	−0.37	0.72	−0.19
	Positive	4	20.01	9.38			
IL-2	Negative	51	14.63	36.84	−3.19	0.002 *	−1.66
	Positive	4	117.38	212.77			

N = number. Ẋ = average. SD = standard deviation. * = statistically significant differences (*p* < 0.05).

**Table 8:** Association effect of interleukins 1β, 2, and 10 on thrombotic events for hospitalized patients with clinical symptoms of COVID-19 in a hospital in the Triângulo Mineiro region, Brazil.

Thrombotic Event (Yes vs. No)				
Predictor	CI (95%)			
	OR	IL	UL	*p*-Value
IL-1β				
pg/mL	1.006	0.999	1.013	0.059
IL-2				
pg/mL	1.01	0.999	1.021	0.061
IL-10				
pg/mL	1.01	1.001	1.011	0.03 *

CI = confidence interval. OR = odds rate. IL = inferior limit. UL = upper limit. Pg = picogram. mL = milliliter. * *p* < 0.05.

## Data Availability

The datasets generated during and/or analysed during the current study are available from the corresponding author on reasonable request.
